# Impact of low serum iron on treatment outcome of PD-1 inhibitors in advanced gastric cancer

**DOI:** 10.1186/s12885-023-11620-9

**Published:** 2023-11-10

**Authors:** Yu Yang, Ya Li, Zhendong Chen

**Affiliations:** 1 Department of Oncology, The Second Affiliated Hospital of Anhui Medical University, No. 678 Fu Rong Road, Hefei, 230601 China; 2 Department of Oncology, Anhui Medical University, Hefei, 230000 China

**Keywords:** Gastric cancer, Serum iron, Immune checkpoint inhibitor, Biomarker

## Abstract

**Background:**

The aim of this study was to investigate the influence of serum iron levels in advanced gastric cancer (GC) patients treated with programmed cell death protein-1 (PD-1) inhibitors.

**Methods:**

We retrospectively reviewed 149 GC patients who were treated with PD-1 inhibitors at our center. Clinicopathological characteristics, laboratory data, and clinical outcomes were analyzed.

**Results:**

Multivariate analysis showed that Eastern Cooperative Oncology Group performance status (ECOG PS), histological subtype, and baseline serum iron levels were independent prognostic factors for overall survival (OS), while ECOG PS, multiple metastatic sites, and baseline serum iron levels were independent prognostic factors for progression-free survival (PFS). Patients with baseline low serum iron levels (LSI) had a significantly shorter median OS and PFS compared to patients with normal serum iron levels (NSI) (Median OS: 7 vs. 14 months, *p* = 0.001; median PFS: 3 vs. 5 months, *p* = 0.005). Patients with baseline LSI had a disease control rate (DCR) of 58.3% at 2 months after PD-1 inhibitor initiation (M2), compared to 81.1% in patients with NSI (*p* = 0.005). Patients with baseline LSI had a DCR of 43.8% at 4 months, compared to 64.2% in patients with NSI (*p* = 0.017).

**Conclusions:**

LSI was associated with worse OS, PFS, and DCR in GC patients treated with PD-1 inhibitors and might be a quick and efficient biomarker to predict the efficacy of PD-1 inhibitors.

**Supplementary Information:**

The online version contains supplementary material available at 10.1186/s12885-023-11620-9.

## Introduction

Gastric cancer (GC) is the fifth most common cancer worldwide and the fourth most common cause of cancer-related death [1]. Iron deficiency (ID) is common in GC patients due to bleeding, diminished oral iron intake, and malabsorption from tumor location. Iron is an essential micronutrient that has a vital role in many biological functions, such as cancer biology and immune functions [2, 3]. ID is one of the most common nutritional deficiencies observed in hospitalized patients, and it is probably highly underestimated. The prevalence of ID in cancer patients ranges from 29–46% [3, 4]. In patients with advanced GC, the reported rate is even higher, ranging from 43.6–78.3% [4, 5]. To date, although some previous studies have investigated and reviewed that ID is associated with the development and prognosis of malignancy [2, 3, 5], the effects of ID have been largely neglected. This certainly deserves more research, since ID occurs particularly frequently in patients with GC, especially advanced GC.

Iron is necessary for the correct functioning of the immune cells [2]. Aly et al. found a significant positive correlation between serum iron level and absolute neutrophil count (*p* = 0.02), CD4% (*p* = 0.001) and CD4/CD8 ratio (*p* = 0.002), and a significant negative correlation between serum iron level and CD19 count (*p* = 0.02) [6]. Iron deficiency anemia (IDA) patients showed a significant reduction in the count and the percentage of CD3 and CD4 [6]. The impairment of immune cells due to ID may result in impaired immunosurveillance and immune microenvironment, which leads to a diminished immune response and, consequentially, an impaired treatment response, a poor prognosis, and reduced overall survival [2, 5]. ID has been analyzed in colorectal cancer patients treated with surgery, and ID was found to be associated with a worse outcome [7, 8]. However, little is known about the influence of ID in advanced GC patients on the effectiveness of programmed cell death protein-1 (PD-1) inhibitors. We sought to investigate the association of serum iron levels with treatment outcomes in advanced GC patients treated with anti-PD-1 therapy.

## Materials and methods

### Patients

We retrospectively analyzed data from 179 consecutive GC patients who were treated with PD-1 inhibitors at the Second Affiliated Hospital of Anhui Medical University (Hefei, China) between September 2019 and September 2021. The study was approved by our institutional review board. Clinicopathological characteristics (including age, sex, histological subtype, stage, tumor location, Eastern Cooperative Oncology Group performance status [ECOG PS]), laboratory data (including serum iron, count of red blood cells, hemoglobin, mean corpuscular volume [MCV], count of white blood cells, count and percentage of neutrophils and lymphocytes), treatments, and clinical outcomes were reviewed. Laboratory data was collected at three different time points: before PD-1 inhibitor initiation (baseline M0), 2 months after PD-1 inhibitor initiation (M2), and 4 months after PD-1 inhibitor initiation (M4).

Low serum iron (LSI) was defined as a serum iron level < 10.6 μmol/L in men and < 7.8 μmol/L in women, which is a commonly used laboratory cut point in our center and refers to Reference Intervals for Common Clinical Biochemistry Tests (WS/T 404.6) in China. Anemia was defined as a hemoglobin level < 130 g/L in men and < 120 g/L in women, according to World Health Organization criteria [9].

The cohort was divided into 4 groups according to M0 and M2 serum iron levels: ①M0–M2– group: LSI at M0 and M2; ②M0–M2 + group: LSI at M0 and normal serum iron level (NSI) at M2; ③M0 + M2– group: NSI at M0 and LSI at M2; ④M0 + M2 + group: NSI at M0 and M2.

### Statistical analysis

The primary objective was to investigate whether baseline serum iron level was associated with overall survival (OS), and secondary endpoints included progression-free survival (PFS) and disease control rate (DCR). OS was defined as the time interval between PD-1 inhibitor initiation and the date of death or last follow-up. PFS was defined as the time interval between PD-1 inhibitor initiation and progression or last follow-up. Progression was determined according to Response Evaluation Criteria in Solid Tumors (RECIST) version 1.1 guidelines [10]. DCR was defined as the proportion of patients with the best overall response of complete response (CR), partial response (PR), or stable disease (SD), per the RECIST version 1.1 guidelines [10].

Clinicopathological characteristics and DCR were compared between patients with and without LSI using the chi-square test. Laboratory data was presented as median (quartile) in the table and was analyzed by rank sum test. Survival curves were plotted using the Kaplan-Meier method and compared by the log-rank test. Prognostic factors that significantly influenced survival in the univariate analysis were included in the Cox proportional hazards model for the multivariate analysis. Statistical analyses were conducted using Statistical Package for the Social Sciences for Windows, software version 25.0 (SPSS Inc., Chicago, IL, USA). All tests were two-sided and *p* < 0.05 was considered statistically significant.

## Results

### Patients’ clinicopathological characteristics

We retrospectively analyzed data from 149 GC patients who were treated with PD-1 inhibitors, excluding 30 patients who were lost to follow-up. Patients’ clinicopathological characteristics at the initiation of anti-PD-1 therapy, both of all patients (n = 149) and separated by serum iron status, are listed in Table 1. There was a male preponderance (n = 113; 75.8%) with a male-to-female ratio of 3.14:1. The median age at the initiation of PD-1 inhibitors was 64 (range, 22–84) years, and the median ECOG PS was 1 (range, 0–3). According to the ECOG PS, 29 patients were 0, 96 patients were 1, 16 patients were 2, and 8 patients were 3. The histological subtype of all 149 patients was adenocarcinoma. The majority of patients (n = 106; 71.1%) were diffuse-type gastric cancer, while 40 patients were intestinal-type and 3 patients were mixed. Human epidermal growth factor 2 (HER-2) status of 81 patients was available, while 18 patients (12.1%) were with HER-2 amplification. Of all 149 patients, 34 (22.8%) had programmed death-ligand 1 (PD-L1) testing on tumor samples and 8 (5.4%) patients underwent mismatch repair (MMR) protein expression testing by immunohistochemistry. Tumor PD-L1 expression using immunohistochemistry was confirmed positive (any level of staining) in 21 patients and negative in 13. Only one patient was identified with the MMR abnormality. The primary tumors of 46 patients (30.9%) were at the gastroesophageal junction (GEJ), while 103 patients (69.1%) were at the stomach. According to the 8th edition of AJCC TNM staging [11], the majority of patients (n = 136; 91.3%) were in stage IV, while one patient was in stage IIA, 8 patients were in stage IIIA, and 4 patients were in stage IIIB. A majority of patients (n = 121; 81.2%) had multiple metastases, while 15 patients developed solitary metastasis and 13 patients had no metastasis.

**Table 1:** Patient and disease characteristics at the initiation of PD-1 inhibitors

Characteristics, n (%)	All patients (n = 149)	LSI (n = 96)	NSI (n = 53)	*p* value
Median age (range)	64 (22–84)	65 (22–84)	64 (30–84)	0.501
< 65 years	76 (51.0)	47 (49.0)	29 (54.7)	
≥ 65 years	73 (49.0)	49 (51.0)	24 (45.3)	
Sex				0.633
Male	113 (75.8)	74 (77.1)	39 (73.6)	
Female	36 (24.2)	22 (22.9)	14 (26.4)	
Primary tumor location				0.005*
GEJ	46 (30.9)	22 (22.9)	24 (45.3)	
Stomach	103 (69.1)	74 (77.1)	29 (54.7)	
Stage				0.081
I-III	13 (8.7)	5 (5.2)	8 (15.1)	
IV	136 (91.3)	91 (94.8)	45 (84.9)	
Histological subtype				0.932
Diffuse	106 (71.1)	69 (71.9)	37 (69.8)	
Intestinal	40 (26.8)	25 (26.0)	15 (28.3)	
Mixed	3 (2.0)	2 (2.1)	1 (1.9)	
HER-2				0.197
Positive	18 (22.2)	9 (17.6)	9 (30.0)	
Negative	63 (77.8)	42 (82.4)	21 (70.0)	
ECOG PS				0.009*
0	29 (19.5)	13 (13.5)	16 (30.2)	
1	96 (64.4)	62 (64.6)	34 (64.2)	
2	16 (10.7)	13 (13.5)	3 (5.7)	
3	8 (5.4)	8 (8.3)	0 (0)	
Anemia				< 0.001*
Yes	121 (81.2)	88 (91.7)	33 (62.3)	
No	28 (18.8)	8 (8.3)	20 (37.7)	
Metastatic sites				0.058
0	13 (8.7)	5 (5.2)	8 (15.1)	
1	15 (10.1)	8 (8.3)	7 (13.2)	
≥ 2	121 (81.2)	83 (86.5)	38 (71.7)	
Surgery				0.499
Yes	62 (41.6)	38 (39.6)	24 (45.3)	
No	87 (58.4)	58 (60.4)	29 (54.7)	
No. of systemic therapy				0.923
1	93 (62.4)	60 (62.5)	33 (62.3)	
2	26 (17.5)	16 (16.7)	10 (18.9)	
≥ 3	30 (20.1)	20 (20.8)	10 (18.9)	
Treatment				0.934
PD-1 monotherapy	15 (10.1)	10 (10.4)	5 (9.4)	
PD-1 + chemo	97 (65.1)	63 (65.6)	34 (64.2)	
PD-1 + TT	33 (22.1)	21 (21.9)	12 (22.6)	
PD-1 + chemo + TT	4 (2.7)	2 (2.1)	2 (3.8)	

Abbreviations: PD-1, programmed cell death protein-1; LSI, low serum iron; NSI, normal serum iron; GEJ, gastroesophageal junction; HER-2, human epidermal growth factor-2; ECOG, Eastern Cooperative Oncology Group; PS, performance status; TT, targeted therapy

*p < 0.05

A total of 96 patients (64.4%) had LSI (baseline serum iron level < 10.6 μmol/L in men and < 7.8 μmol/L in women) at the initiation of PD-1 inhibitors. The median serum iron levels of the patient population were 8.40 μmol/L (range: 1.1–22.4 μmol/L) in men and 7.0 μmol/L (range: 2.3–30.1 μmol/L) in women. A total of 121 patients (81.2%) had anemia at the initiation of PD-1 inhibitors. The median counts of red blood cells and hemoglobin of the patient population were 3.81 × 10^12^/L (range: 1.78 × 10^12^ – 5.38 × 10^12^/L) and 110 g/L (range: 35–161 g/L) in men, and 3.72 × 10^12^/L (range: 2.50 × 10^12^ – 4.67 × 10^12^/L) and 102 g/L (range: 78–136 g/L) in women, respectively. Patients who had baseline LSI were more likely to have worse ECOG PS (*p* = 0.009), primary tumors at the stomach (*p* = 0.005), and anemia (*p* < 0.001) (Table 1).

**Fig. 1:**
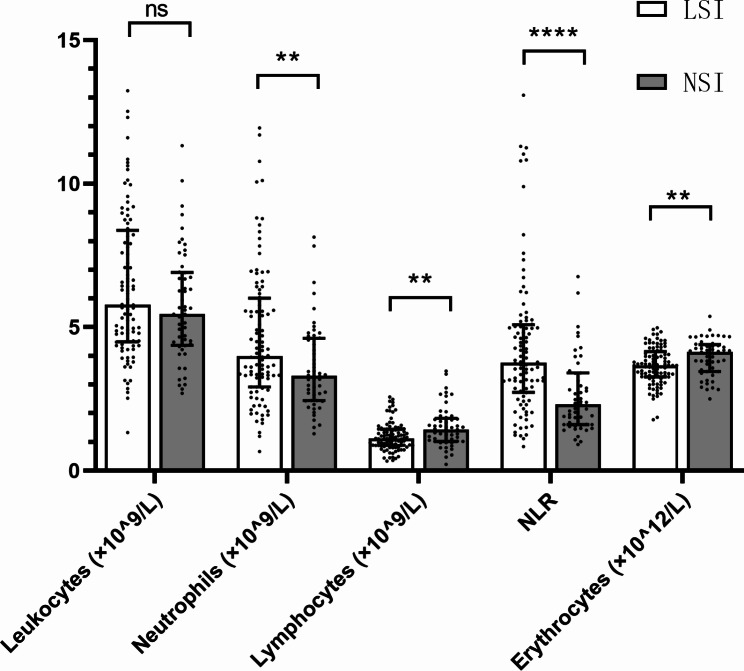
Comparison of total blood count parameter between low serum iron (LSI) group and normal serum iron (NSI) group. Data shows median values ± quartile. ** *p* < 0.01, **** *p* < 0.0001, ns: not significant, NLR: neutrophil-to-lymphocyte ratio

Regarding leukocytes, there were no significant differences in the white blood cell counts between groups. However, the counts and proportion of neutrophils were higher in the LSI group than in the NSI group (3.99 × 10^9^/L vs. 3.30 × 10^9^/L, *p* = 0.008; 69.98% vs. 61.79%, *p* < 0.001), concomitantly with a decrease in the counts and proportion of lymphocytes (1.12 × 10^9^/L vs. 1.44 × 10^9^/L, *p* = 0.001; 18.55% vs. 27.32%, *p* < 0.001)(Table 2; Fig. 1). Regarding erythrocytes, the red blood cell counts, hemoglobin and MCV were lower in the LSI group than in the NSI group (Table 2).

**Table 2:** Total blood count parameter comparison between groups

Characteristics, median (quartile)	LSI (n = 96)	NSI (n = 53)	*p* value
Leukocytes (×10^9^/L)	5.79 (4.49, 8.38)	5.45 (4.37, 6.91)	0.165
Neutrophils (×10^9^/L)	3.99 (2.92, 6.01)	3.30 (2.45, 4.61)	0.008*
Neutrophils (%)	69.98 (63.26, 75.78)	61.79 (53.12, 67.98)	< 0.001*
Lymphocytes (×10^9^/L)	1.12 (0.88, 1.45)	1.44 (1.03, 1.83)	0.001*
Lymphocytes (%)	18.55 (14.47, 23.34)	27.32 (20.89, 32.70)	< 0.001*
NLR	3.77 (2.73, 5.08)	2.31 (1.61, 3.40)	< 0.001*
Erythrocytes (×10^12^/L)	3.67 (3.27, 4.15)	4.13 (3.46, 4.39)	0.007*
Hemoglobin (g/L)	101 (92, 114.75)	122 (106.5, 132)	< 0.001*
MCV (fL)	88.45 (83.83, 94.25)	92.60 (88.55, 96.10)	0.002*

Abbreviations: LSI, low serum iron; NSI, normal serum iron; NLR, neutrophil-to-lymphocyte ratio; MCV, mean corpuscular volume

*p < 0.05

### Treatments

Among the 149 patients in our study, 62 patients (41.6%) underwent surgical resections of the primary tumor before. Patients received one of the following PD-1 inhibitors: camrelizumab (59.1%), tislelizumab (16.1%), sintilimab (13.4%), toripalimab (8.7%), pembrolizumab (1.3%), or nivolumab (1.3%). The majority of patients (n = 93; 62.4%) received anti-PD-1 therapy as first-line treatment, 26 as second-line treatment, and 30 as third-line or above treatment. There were 15 patients receiving anti-PD-1 monotherapy, 97 receiving anti-PD-1 therapy in combination with chemotherapy, 33 receiving anti-PD-1 therapy in combination with targeted therapy, and 4 receiving anti-PD-1 therapy in combination with chemotherapy and targeted therapy. The chemotherapy regimen mainly consisted of platinum-based, fluorouracil-based, or paclitaxel-based drugs. The targeted therapy was apatinib.

### Survival analyses

The median follow-up time was 29 (range, 1–57) months. At the time of the last follow-up, 114 patients (76.5%) died of cancer. The median survival time of all 149 patients was 9 (95% confidence interval [CI]: 8.021–11.979) months. The 1- and 2-year OS rates after PD-1 inhibitors initiation were 39.6% and 22.1%, respectively. In the univariate analysis, the age, sex, primary tumor location, Her-2 status, PD-L1, surgery, and treatment strategies of PD-1 inhibitors showed no significant effects on OS, while histological subtype, stage, metastatic sites, ECOG PS, baseline serum iron levels and treatment lines were significantly associated with OS (Supplementary Table 1). The age, sex, Her-2 status, PD-L1, surgery, and treatment strategies of PD-1 inhibitors showed no significant effects on PFS, while primary tumor location, histological subtype, stage, metastatic sites, ECOG PS, baseline serum iron levels, and treatment lines were significantly associated with PFS (Supplementary Table 1). Multivariate analysis showed that ECOG PS, histological subtype, and baseline serum iron levels were independent prognostic factors for OS, while ECOG PS, metastatic sites, and baseline serum iron levels were independent prognostic factors for PFS (Table 3).

**Table 3:** Multivariate analysis of prognostic factors associated with overall survival (OS) and progression-free survival (PFS) in advanced gastric cancer patients

Parameter	PFS		OS	
	HR (95%CI)	*p*-value	HR (95%CI)	*p*-value
Baseline serum iron levels LSI vs. NSI	0.672 (0.462–0.977)	0.038*	0.562 (0.368–0.859)	0.008*
Histological subtype Diffuse vs. Intestinal	0.730 (0.490–1.085)	0.120	0.630 (0.397–0.999)	0.0497*
ECOG PS 0–1 vs. ≥2	2.208 (1.335–3.651)	0.002*	2.828 (1.663–4.809)	< 0.001*
Stage I-III vs. IV	0.765 (0.329–1.779)	0.533	1.056 (0.373–2.993)	0.918
Primary tumor location GEJ vs. Stomach	1.280 (0.876–1.870)	0.202	-	-
Metastatic sites 0–1 vs. ≥2	2.181 (1.135–4.191)	0.019*	1.918 (0.950–3.874)	0.069
No. of systemic therapy 1 vs. ≥2	1.272 (0.880–1.837)	0.200	1.348 (0.898–2.024)	0.150

Abbreviations: OS, overall survival; PFS, progression-free survival; HR, hazard ratio; CI, confidence interval; LSI, low serum iron; NSI, normal serum iron; ECOG, Eastern Cooperative Oncology Group; PS, performance status; GEJ, gastroesophageal junction

*p < 0.05

### Association between serum iron level and prognosis in 149 patients

**Fig. 2:**
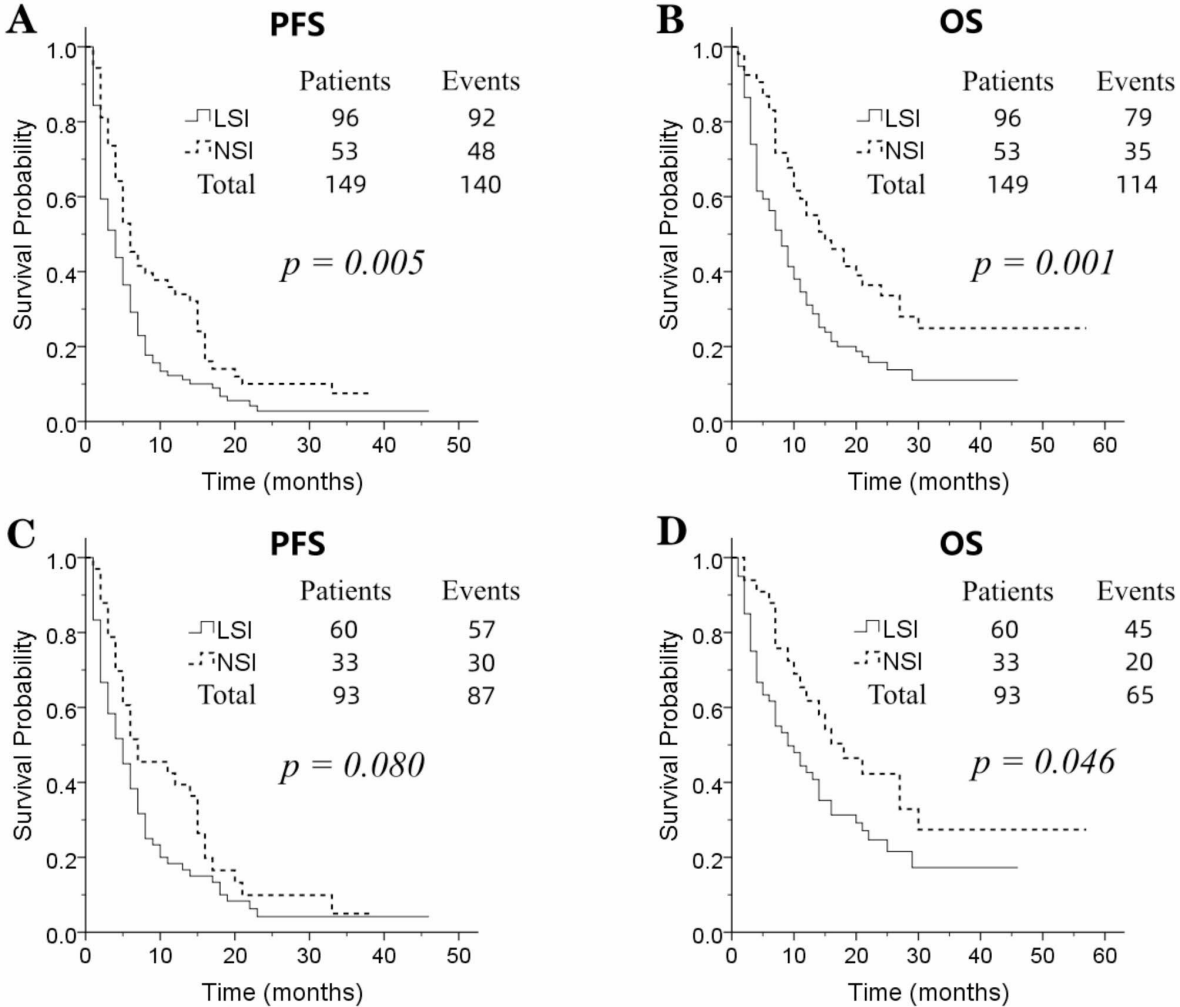
(**A**) The progression-free survival (PFS) curves between low serum iron (LSI) and normal serum iron (NSI) in all 149 gastric cancer patients; (**B**) the overall survival (OS) curves between LSI and NSI in all 149 gastric cancer patients; (**C**) the PFS curves between LSI and NSI in 93 patients receiving first-line treatment; (**D**) the OS curves between LSI and NSI in 93 patients receiving first-line treatment

When evaluating OS and PFS, we observed that patients with baseline LSI had a significantly shorter median OS (mOS) and median PFS (mPFS) compared to patients with NSI (mOS: 7 vs. 14 months, *p* = 0.001; mPFS: 3 vs. 5 months, *p* = 0.005) (Fig. 2A, B).

We divided the patients into 3 groups according to the regimens of anti-PD-1-containing therapy: the anti-PD-1 monotherapy group, the anti-PD-1 plus chemotherapy group, and the anti-PD-1 plus targeted therapy group. We found that in the anti-PD-1 + chemotherapy group and the anti-PD-1 + targeted therapy group, LSI had worse PFS and OS compared to NSI (The anti-PD-1 + chemotherapy group: mOS: 8 vs. 18 months, *p* = 0.020; mPFS: 4 vs. 7 months, *p* = 0.049; the anti-PD-1 + targeted therapy group: mOS: 4 vs. 10 months, *p* = 0.003; mPFS: 2 vs. 6 months, *p* = 0.013). No statistically significant differences were observed in the anti-PD-1 monotherapy group, however, there was a tendency for LSI to have a worse prognosis than NSI (Supplementary Fig. 1).

The associations between baseline serum iron level and treatment response to PD-1 inhibitors using RECIST criteria showed that LSI was significantly associated with a lower DCR (Table 4). Patients with baseline LSI had a DCR of 58.3% at 2 months compared to 81.1% in patients with NSI (odds ratio [OR]: 3.071; 95% CI: 1.382–6.828; *p* = 0.005), and a DCR of 43.8% at 4 months compared to 64.2% in patients with NSI (OR: 2.301; 95% CI: 1.153–4.593; *p* = 0.017).

**Table 4:** Response to PD-1 inhibitors and association with baseline serum iron levels

Time of Evaluation	Response to treatment	All patients	LSI	NSI	*p* value
M2	CR	0	0	0	0.003*
	PR	36	18	18	
	SD	63	38	25	
	PD	50	40	10	
	ORR	24.2%	18.8%	34.0%	
	DCR	66.4%	58.3%	81.1%	0.005*
M4	CR	0	0	0	0.011*
	PR	34	17	17	
	SD	42	25	17	
	PD	73	54	19	
	ORR	22.8%	17.7%	32.1%	
	DCR	51.0%	43.8%	64.2%	0.017*

Abbreviations: PD-1, programmed cell death protein-1; LSI, low serum iron; NSI, normal serum iron; CR, complete response; PR, partial response; SD, stable disease; PD, progressive disease

*p < 0.05

**Fig. 3:**
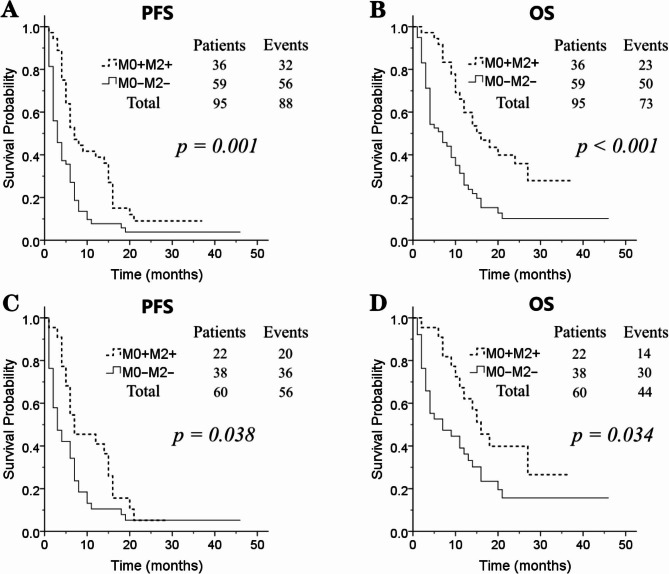
(**A**) The progression-free survival (PFS) curves between M0–M2– group and M0 + M2 + group in all 149 gastric cancer patients; (**B**) the overall survival (OS) curves between M0–M2– group and M0 + M2 + group in all 149 gastric cancer patients; (**C**) the PFS curves between M0–M2– group and M0 + M2 + group in 93 patients receiving first-line treatment; (**D**) the OS curves between M0–M2– group and M0 + M2 + group in 93 patients receiving first-line treatment (M0: baseline serum iron level; M2: serum iron level at 2 months after PD-1 inhibitor initiation; M0–M2– group: low serum iron at M0 and M2; M0 + M2 + group: normal serum iron at M0 and M2)

According to the serum iron level fluctuation pattern, the serum iron levels of 143 patients were available and were divided into 4 groups: 59 patients in the M0–M2–group, 32 in the M0–M2 + group, 16 in the M0 + M2– group, and 36 in the M0 + M2 + group. The mPFS of the four groups were 2 months, 5 months, 3 months, and 7 months, respectively (*p* = 0.011). The mOS of the four groups were 6 months, 9 months, 7 months, and 15 months, respectively (*p* = 0.002). The 1-year OS rates were 25.8%, 42.8%, 41.7%, and 59.8%, respectively. Patients in the M0–M2– group showed a significant decrease in OS and PFS compared to patients in the M0 + M2 + group (mOS: 6 vs. 15 months, *p* < 0.001; mPFS: 3 vs. 6 months, *p* = 0.001) (Fig. 3A, B). Patients in the M0–M2– group had a 1.34-fold higher risk of death than patients in the M0 + M2 + group (OR: 1.343; 95% CI: 1.136–1.588; *p* < 0.001).

### Association between serum iron level and prognosis in 93 patients receiving first-line treatment

In 93 patients receiving first-line treatment, 65 patients (69.9%) died of cancer. The median survival time of 93 patients was 12 (95% CI: 8.601–15.399) months. The 1- and 2-year OS rates after PD-1 inhibitors initiation were 49.4% and 29.0%, respectively. Univariate analysis showed that baseline serum iron level was significantly associated with OS but not PFS (mOS: 9 vs. 16 months, *p* = 0.046; mPFS: 4 vs. 6 months, *p* = 0.080) (Fig. 2C, D).

Patients with baseline LSI had a DCR of 66.7% at 2 months compared to 87.9% in patients with NSI (OR: 3.625; 95% CI: 1.119–11.740; *p* = 0.025), and a DCR of 55.0% at 4 months compared to 78.8% in patients with NSI (OR: 3.039; 95% CI: 1.144–8.076; *p* = 0.023) (Supplementary Table 2).

According to the serum iron level fluctuation pattern, the serum iron levels of 92 patients were available and were divided into 4 groups: 38 patients in the M0–M2–group, 21 in the M0–M2 + group, 11 in the M0 + M2– group, and 22 in the M0 + M2 + group. The mOS of the four groups were 5 months, 10 months, 21 months, and 15 months, respectively. The 1-year OS rates were 36.2%, 51.6%, 63.6%, and 62.1%, respectively. Patients in the M0–M2– group showed a decreasing trend in OS and PFS compared to patients in the M0 + M2 + group (mOS: 7 vs. 15 months, *p* = 0.034; mPFS: 3 vs. 7 months, *p* = 0.038) (Fig. 3C, D).

## Discussion

ID is one of the most frequent hematological manifestations in individuals with cancer, and is especially common in patients with GC. The prevalence of ID in GC may be underestimated due to bleeding, diminished oral iron intake, malabsorption from tumor location, and surgical excision. In a Canadian retrospective study, 56% of GC patients were diagnosed with ID, and 40% had IDA [4]. In our study, 81.2% of GC patients were diagnosed with anemia, and 64.4% (96/149) had LSI.

To our knowledge, this is the first study to evaluate the association of serum iron level with the outcome of GC patients treated with PD-1 inhibitors. LSI was found to be associated with a significantly lower OS, PFS, and DCR in GC patients receiving anti-PD-1 therapy. Patients with LSI had 5.8% and 24.6% increases in the risk of treatment failure or death, respectively. In addition, the 31.8% reduction in the odds of disease control suggested that tumors with baseline LSI were more likely to be more aggressive or have a worse treatment response to anti-PD-1 therapy.

Iron is critical in maintaining the immune system by regulating the growth and differentiation of immune cells [2]. The ID resulted in impaired cellular immunity, especially leading to defects in T-cell maturation, halting of macrophage differentiation and impaired NKC activity [12]. ID also decreased T-cell proliferation and production of cytokines and, consequentially, led to a deficiency in cell-mediated immune responses [2, 12–14]. Our results showed a significantly decreased count (22.2% lower) and percentage (32.1% lower) of circulating lymphocytes in the LSI group. A decreased count and percentage of lymphocytes may induce impaired cellular immunity in LSI patients. Many previous studies have shown that PD-1 inhibitors enhance the T cell response and mediate antitumor activity by blocking the interaction between PD-1 and PD‐L1 [15, 16]. Because PD-1 inhibitors enhanced antitumor immunity by blocking negative regulators of T cell function, it was plausible that alterations in the relative proportions of circulating lymphocytes could influence the efficacy of PD-1 inhibitors. Previous studies showed that, in patients treated with PD-1 inhibitors, inferior outcomes were associated with elevated neutrophil-to-lymphocyte ratio (NLR) [17, 18]. Low levels of circulating lymphocytes might result in a weakened lymphocyte-mediated immune response. Similarly, high levels of circulating neutrophils could inhibit lymphocyte-mediated antitumor activity and release various inflammatory cytokines that promote cancer progression. In our study, LSI was prone to developing high NLR, with the possibility of an impaired treatment response to anti-PD-1 therapy. Unfortunately, we did not assess the functional phenotype of lymphocytes. Further analysis of lymphocyte subtypes, ID and PD-1 inhibitors is recommended.

ID resulted in an insufficient immunosurveillance ability of the immune system, which, in turn, created a favorable condition for the development and progression of cancer [2]. Some previous studies determined that patients with IDA had an increased risk of cancer development [19, 20]. In addition, patients with IDA had inferior outcomes and presented with worse tumor staging and lower disease-free survival compared with those without IDA [7, 8]. Our results showed that LSI was associated with unfavorable prognostic features, such as poor ECOG PS and primary tumor located in the stomach, which were similar to those observed in previous research reports [21, 22]. Collectively, these mechanisms revealed that tumors with baseline LSI were more likely to be more aggressive, which might explain why GC patients with LSI had significantly lower OS, PFS, and DCR when receiving anti-PD-1 therapy. In addition, our results showed that patients in the M0–M2– group had a 1.34-fold higher risk of death than patients in the M0 + M2 + group. This suggested that the occurrence or persistence of LSI represented a marker of unfavorable prognosis for GC patients treated with PD-1 inhibitors.

In our study, LSI had worse PFS and OS compared to NSI in the anti-PD-1 plus chemotherapy group and the anti-PD-1 plus targeted therapy group. No statistically significant differences were observed in the anti-PD-1 monotherapy group, however, there was a tendency for LSI to have a worse prognosis than NSI. One possible reason for this result was the small number of patients (only 15) in the anti-PD-1 monotherapy group. Furthermore, previous studies reported a transient increase in serum iron levels following chemotherapy, such as leucovorin and fluorouracil plus oxaliplatin (FOLFOX), leucovorin and fluorouracil plus irinotecan (FOLFIRI), actinomycin D, adriamycin, and cyclophosphamide [23, 24]. However, the serum iron level was transiently elevated after chemotherapy (48 h), returning to the initial level within two weeks [23, 24]. Follézou and Bizon observed a decrease in reticulocytes in parallel with an elevated serum iron level, suggesting that chemotherapy drugs damaged reticuloendothelial cells involved in iron metabolism [23]. Ochiai et al. proposed that the elevation of serum iron levels during chemotherapy may be secondary to reduced iron consumption by erythropoiesis [25]. The targeted drug had no effect on serum iron levels [26]. Since the aim of this study was to investigate the association between baseline serum iron level and prognosis, baseline LSI was associated with a poorer prognosis, regardless of PD-1 inhibitors in combination with chemotherapy or PD-1 inhibitors in combination with targeted therapy, which reflected the relationship between baseline serum iron level and the prognosis of anti-PD-1 therapy.

Although PD-1 inhibitors have shown promising results in phase I to III clinical trials in GC, it is noteworthy that not all patients could get benefits [27–29]. Therefore, it was necessary to select the appropriate predictive factors and screen the target population before anti-PD-1 therapy. Up to now, it had been proved that the expression rate of PD-L1, microsatellite instability and tumor mutation burden were reliable predictive biomarkers for the treatment effects of PD-1 inhibitors [29–33]. However, these markers were difficult to detect and costly. Serum iron levels could be easily determined via routine plasma electrolyte tests. Hence, it could be repeated and sequentially studied. Our study revealed that LSI was correlated with poor treatment response and an unfavorable prognosis for GC patients treated with PD-1 inhibitors. The ease of use and low cost of this marker warrant its further evaluation in future studies. In addition, whether supplementing iron during anti-PD-1 treatment for LSI patients improved efficacy also warranted further prospective studies with larger cohorts.

Our study had some limitations, which included the retrospective nature of its design and the lack of total iron binding capacity (TIBC), transferrin saturation (TSAT), and ferritin. Moreover, PD-1 expression status was available in very few patients, and we, therefore, could not include this as a potential variable in our analyses. Lastly, it is unknown if aggressive treatment of baseline low serum iron could eventually alter the outcome of GC patients treated with anti-PD-1 therapy. However, despite these limitations and biases, this is the only study to our knowledge to assess serum iron levels in GC patients treated with anti-PD-1 therapy.

In conclusion, we have shown that LSI was associated with poorer OS, PFS, and DCR in GC patients treated with PD-1 inhibitors. Serum iron levels may be a quick and efficient marker to predict the efficacy of PD-1 inhibitors and deserve to be further investigated as a prognostic factor.

### Electronic supplementary material

Below is the link to the electronic supplementary material.

Supplementary Material 1: Fig. 1 The progression-free survival (PFS) curves between low serum iron (LSI) and normal serum iron (NSI) in the anti-PD-1 monotherapy group (A), the anti-PD-1 plus chemotherapy group (B), and the anti-PD-1 plus targeted therapy group (C). The overall survival (OS) curves between LSI and NSI in the anti-PD-1 monotherapy group (D), the anti-PD-1 plus chemotherapy group (E), and the anti-PD-1 plus targeted therapy group (F)

Supplementary Material 2: Table 1 Univariate analysis of progression-free survival (PFS) and overall survival (OS) in gastric cancer

Supplementary Material 3: Table 2 Response to PD-1 inhibitors and association with baseline serum iron levels in 93 patients receiving first-line treatment

## Data Availability

The data used to support the findings of this study are restricted by the review board of the Second Affiliated Hospital of Anhui Medical University in order to protect patient privacy. Data are available from Yu Yang (E-mail: 15055127520@163.com) for researchers who meet the criteria for access to confidential data.

## Abbreviations

CI: confidence interval
CR: complete response
DCR: disease control rate
ECOG PS: Eastern Cooperative Oncology Group performance status
GC: Gastric cancer
GEJ: gastroesophageal junction
HER-2: Human epidermal growth factor 2
ID: Iron deficiency
IDA: iron deficiency anemia
LSI: Low serum iron
MCV: mean corpuscular volume
MMR: mismatch repair
NLR: neutrophil-to-lymphocyte ratio
NSI: normal serum iron level
OR: odds ratio
OS: overall survival
PD-1: programmed cell death protein-1
PD-L1: programmed death-ligand 1
PFS: progression-free survival
PR: partial response
RECIST: Response Evaluation Criteria in Solid Tumors
SD: stable disease
TIBC: total iron binding capacity
TSAT: transferrin saturation
