# Supplementary figures and images for: Impact of low serum iron on treatment outcome of PD-1 inhibitors in advanced gastric cancer
Source: BMC Cancer. 2023 Nov 10;23:1095. doi: 10.1186/s12885-023-11620-9 (PMC10638799; doi:10.1186/s12885-023-11620-9)

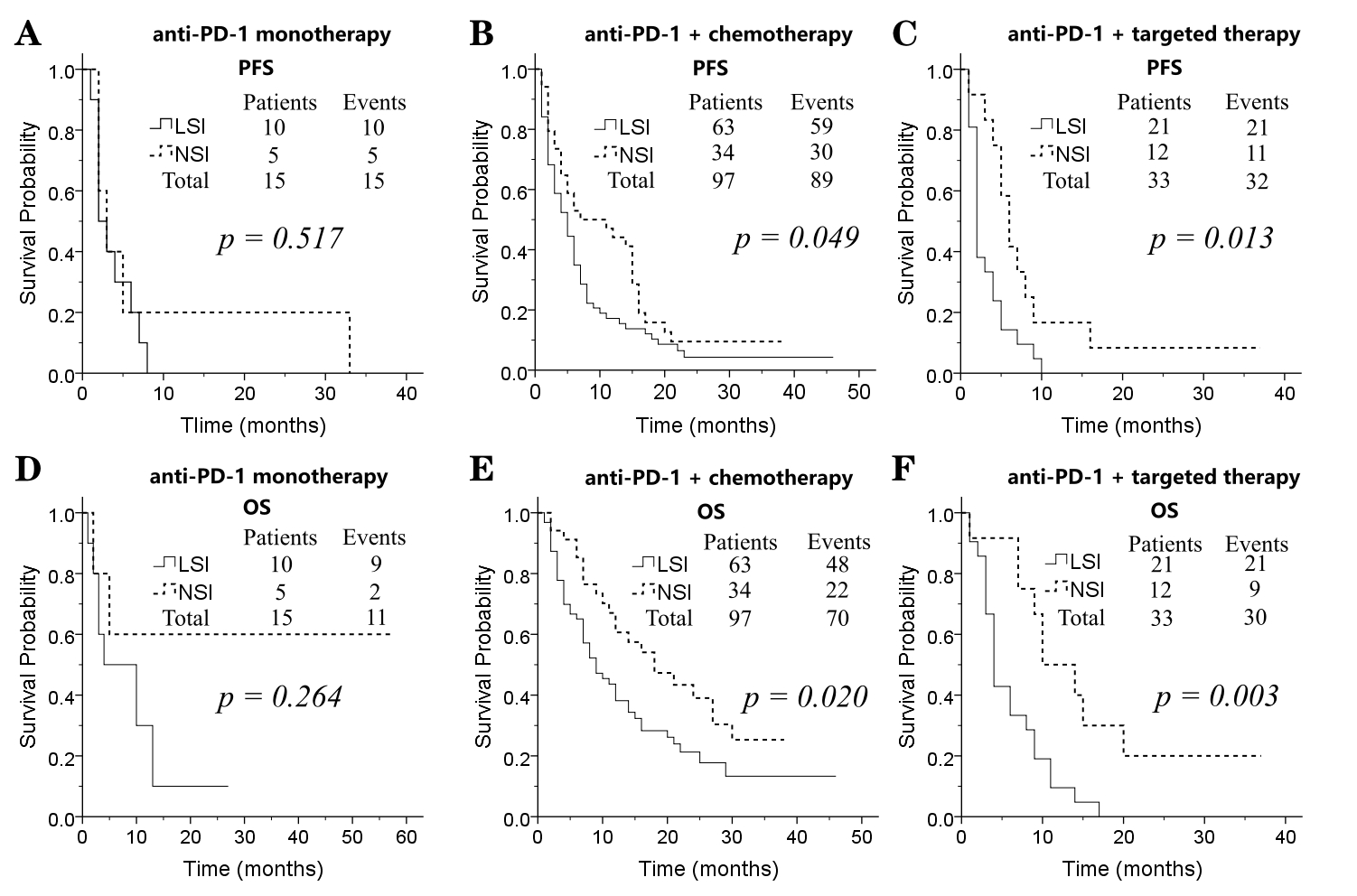

Supplement: Supplementary file 1 — Supplementary Material 1: Fig. 1 The progression-free survival (PFS) curves between low serum iron (LSI) and normal serum iron (NSI) in the anti-PD-1 monotherapy group (A), the anti-PD-1 plus chemotherapy group (B), and the anti-PD-1 plus targeted therapy group (C). The overall survival (OS) curves between LSI and NSI in the anti-PD-1 monotherapy group (D), the anti-PD-1 plus chemotherapy group (E), and the anti-PD-1 plus targeted therapy group (F) [file 12885_2023_11620_MOESM1_ESM.jpg]
