# Supplementary material for: Impact of low serum iron on treatment outcome of PD-1 inhibitors in advanced gastric cancer
Source: BMC Cancer. 2023 Nov 10;23:1095. doi: 10.1186/s12885-023-11620-9 (PMC10638799; doi:10.1186/s12885-023-11620-9)
Supplement: Supplementary file 2 — Supplementary Material 2: Table 1 Univariate analysis of progression-free survival (PFS) and overall survival (OS) in gastric cancer [file 12885_2023_11620_MOESM2_ESM.docx]

Supplementary Table 1 Univariate analysis of progression-free survival (PFS) and overall survival (OS) in gastric cancer

| **Characteristics** | **Patients** | **PFS** | | **OS** | |
| --- | --- | --- | --- | --- | --- |
|  | **n (%)** | **HR(95%CI)** | ***p* value** | **HR(95%CI)** | ***p* value** |
| **Median age** |  | 0.848 (0.608-1.182) | 0.294 | 0.906 (0.627-1.309) | 0.586 |
| <65 years | 76 (51.0) |  |  |  |  |
| ≥65 years | 73 (49.0) |  |  |  |  |
| **Sex** |  | 1.124 (0.768-1.646) | 0.515 | 0.953 (0.618-1.468) | 0.821 |
| Male | 113 (75.8) |  |  |  |  |
| Female | 36 (24.2) |  |  |  |  |
| **Primary tumor location** |  | 1.458 (1.011-2.103) | 0.029* | 1.472 (0.974-2.224) | 0.056 |
| GEJ | 46 (30.9) |  |  |  |  |
| Stomach | 103 (69.1) |  |  |  |  |
| **Stage** |  | 1.875 (1.009-3.485) | 0.030* | 2.634 (1.156-6.004) | 0.013* |
| I-III | 13 (8.7) |  |  |  |  |
| IV | 136 (91.3) |  |  |  |  |
| **Histological subtype** |  | 0.647 (0.441-0.950) | 0.016* | 0.544 (0.348-0.851) | 0.005* |
| Diffuse | 106 (71.1) |  |  |  |  |
| Intestinal | 40 (26.8) |  |  |  |  |
| **HER-2** |  | 1.352 (0.791-2.309) | 0.231 | 1.163 (0.649-2.084) | 0.599 |
| Positive | 18 (12.1) |  |  |  |  |
| Negative | 63 (42.3) |  |  |  |  |
| **ECOG PS** |  | 1.725 (1.350-2.204) | ＜0.001* | 2.058 (1.574-2.689) | ＜0.001* |
| 0 | 29 (19.5) |  |  |  |  |
| 1 | 96 (64.4) |  |  |  |  |
| 2 | 16 (10.7) |  |  |  |  |
| 3 | 8 (5.4) |  |  |  |  |
| **Metastatic sites** |  | 1.747 (1.275-2.393) | ＜0.001* | 1.910 (1.303-2.798) | 0.001* |
| 0 | 13 (8.7) |  |  |  |  |
| 1 | 15 (10.1) |  |  |  |  |
| ≥2 | 121 (81.2) |  |  |  |  |
| **Baseline serum iron levels** |  | 0.627 (0.440-0.894) | 0.005* | 0.536 (0.359-0.802) | 0.001* |
| LSI | 96 (64.4) |  |  |  |  |
| NSI | 53 (35.6) |  |  |  |  |
| **PD-L1** |  | 0.605 (0.292-1.255) | 0.155 | 0.650 (0.309-1.369) | 0.243 |
| Positive | 21 (14.1) |  |  |  |  |
| Negative | 13 (8.7) |  |  |  |  |
| **Surgery** |  | 1.094 (0.778-1.538) | 0.578 | 1.405 (0.969-2.038) | 0.063 |
| Yes | 62 (41.6) |  |  |  |  |
| No | 87 (58.4) |  |  |  |  |
| **No. of systemic therapy** |  | 1.196 (0.978-1.463) | 0.022* | 1.322 (1.064-1.643) | 0.011* |
| 1 | 93 (62.4) |  |  |  |  |
| 2 | 26 (17.5) |  |  |  |  |
| ≥3 | 30 (20.1) |  |  |  |  |
| **Treatment** |  | 1.085 (0.820-1.435) | 0.074 | 1.232 (0.912-1.664) | 0.055 |
| PD-1 monotherapy | 15 (10.1) |  |  |  |  |
| PD-1+chemo | 97 (65.1) |  |  |  |  |
| PD-1+TT | 33 (22.1) |  |  |  |  |
| PD-1+chemo+TT | 4 (2.7) |  |  |  |  |

**p* < 0.05

Abbreviations: PD-1, programmed cell death protein-1; LSI, low serum iron; NSI, normal serum iron; GEJ, gastroesophageal junction; HER-2, human epidermal growth factor-2; ECOG, Eastern Cooperative Oncology Group; PS, performance status; TT, targeted therapy
