# Supplementary material for: Impact of low serum iron on treatment outcome of PD-1 inhibitors in advanced gastric cancer
Source: BMC Cancer. 2023 Nov 10;23:1095. doi: 10.1186/s12885-023-11620-9 (PMC10638799; doi:10.1186/s12885-023-11620-9)
Supplement: Supplementary file 3 — Supplementary Material 3: Table 2 Response to PD-1 inhibitors and association with baseline serum iron levels in 93 patients receiving first-line treatment [file 12885_2023_11620_MOESM3_ESM.docx]

Supplementary Table 2 Response to PD-1 inhibitors and association with baseline serum iron levels in 93 patients receiving first-line treatment

| Time of Evaluation | Response to treatment | All patients | LSI | NSI | *p* value |
| --- | --- | --- | --- | --- | --- |
| M2 | CR | 0 | 0 | 0 | 0.024* |
|  | PR | 32 | 17 | 15 |  |
|  | SD | 37 | 23 | 14 |  |
|  | PD | 24 | 20 | 4 |  |
|  | ORR | 34.4% | 28.3% | 45.5% |  |
|  | DCR | 74.2% | 66.7% | 87.9% | 0.025* |
| M4 | CR | 0 | 0 | 0 | 0.028* |
|  | PR | 30 | 16 | 14 |  |
|  | SD | 29 | 17 | 12 |  |
|  | PD | 34 | 27 | 7 |  |
|  | ORR | 32.3% | 26.7% | 42.4% |  |
|  | DCR | 63.4% | 55.0% | 78.8% | 0.023* |

**p* < 0.05

Abbreviations: PD-1, programmed cell death protein-1; LSI, low serum iron; NSI, normal serum iron; CR, complete response; PR, partial response; SD, stable disease; PD, progressive disease
